# D‐Licensing in Adjectival Passives†

**DOI:** 10.1111/stul.12108

**Published:** 2019-01-13

**Authors:** Peter Hallman

**Affiliations:** ^1^ Austrian Research Institute for Artificial Intelligence Freyung 6/6 1010 Vienna Austria

## Abstract

This article argues that in addition to the familiar Case licensing requirement known as the ‘Case Filter’, nominal arguments (DPs) are subject to a requirement that I refer to as ‘D‐licensing’, construed as a requirement of DPs themselves. It is claimed that this notion of D‐licensing is useful in the characterization of restrictions on adjectival participle formation. Adjectival participles fail to license the full complement frame of the corresponding verb not because they fail to assign Case, but because they lack D‐licensing structure.

## 1 Introduction

In this paper, I claim that patterns of object licensing in verbal and adjectival passive constructions in English and German, as well as in clitic constructions in certain Arabic dialects, point to the conclusion that nominal arguments (‘DPs’ after Abney 1987) are subject to a licensing requirement above and beyond the familiar Case licensing requirement known as the ‘Case Filter’. The Case Filter requires every DP to be associated with a Case position at some point in the derivation (Chomsky 1981, 1986). I claim here that DPs must additionally be associated with a D‐licensing position at some point in the derivation. I refer to this licensing requirement as ‘D‐licensing’, though the notion of D‐licensing that emerges here is not identical to the notion of D‐licensing described by Chomsky (1995), Alexiadou and Anagnostopoulou (1998), Holmberg (2000), Anagnostopoulou (2003), and others. These authors construe D‐licensing, like the EPP, as a requirement of certain syntactic positions. I develop another notion of D‐licensing here in which D‐licensing is a requirement of DPs themselves, such that if a DP fails to enter into a checking relation with an appropriate D‐licensing head in the course of a derivation, the derivation fails. The EPP is a special requirement of the highest D‐licensing position in English, that it overtly attract its licensee. I claim also that in some languages, certain D‐licensing positions are subject to a definiteness requirement. These claims are motivated in the first instance by observations about the distribution of DPs in adjectival participle construction in English, as described in section 2 below. Section 3 describes contemporary analyses of the internal syntax of adjectival participles. Sections 4 and 5 describe in detail the incorrect prediction of such analyses that adjectival participles of double object verbs should be possible, and argue against a Case‐based solution. Section 6 proposes a solution in terms of D‐licensing, while section 7 extends the analysis to German, which presents further evidence against a Case‐based solution. Sections 8 and 9 discuss the connection between D‐licensing and definiteness, while section 10 presents an analysis of certain exceptions to the ban on adjectival participles of double object constructions.

## 2 Verbal and Adjectival Participles

It is characteristic of prototypical passive constructions that the term that occurs as object of the active counterpart occurs as subject of the passive, as shown in (1). In English and many other Indo‐European languages, the main verb occurs as a participle in the passive construction, whose characterizing morphological feature is the suffix *‐en* with allomorphs ‐*ed* and others, supported by the auxiliary *be*.

**Table:** 

(1)	a.	Mary painted the door.
	b.	The door was painted by Mary.

Wasow (1977) identifies a variety of criteria that distinguish what are now usually called ‘verbal’ and ‘adjectival’ passive participles. Verbal participles have the same aspectual profile as the corresponding verb. Insofar as active *paint* may occur in the progressive, for example, its verbal passive counterpart *be painted* may as well. Occurrence in the progressive is characteristic of eventive verbs (Vendler 1957, Vlach 1981, and many others), meaning *be painted* is eventive in (2b).

**Table:** 

(2)	a.	Mary was painting the door.
	b.	The door was being painted by Mary.

Adjectival participles, on the other hand, are systematically stative and occur only in a variety of contexts restricted to adjectives, like the complement of quasi‐auxiliary verbs like *seem* and *remain*.

**Table:** 

(3)	The door seems/remains freshly painted.

These contexts also include the simple present tense in English (4a), where bona fide eventive verbs are excluded (4b).^1^

**Table:** 

(4)	a.	The door is (freshly) painted.
	b.	*Mary (freshly) paints the door.

Wasow observes that in such contexts, passive participles do not readily admit *by*‐phrases, as illustrated in (5a), and become compatible with the adjectival prefix *un‐*, which does not occur with verbs, as shown in (5b).^2^

**Table:** 

(5)	a.	*The door is/seems/remains (freshly) painted by Mary.
	b.	The door is/seems/remains unpainted.

Another attribute that distinguishes verbal and adjectival passive participles is the possibility of co‐reference between the subject and the implicit agent. Baker, Johnson and Roberts (1989) point out that verbal participle constructions like (6a) (in the progressive to rule out an adjectival passive reading) cannot be understood to mean that the children are washing themselves. Kratzer (2000) points out that adjectival participles do not share this property. Example (6b) is compatible with a situation in which the children washed themselves.

**Table:** 

(6)	a.	The children are being washed.
	b.	The children seem (thoroughly) washed.

Lastly, and crucially for the present purposes, Wasow observes that double object constructions like (7a) may form verbal (7b) but not adjectival (7c) passives. In verbal passives, the ‘primary’ object (the first DP following the verb in the active, *the collector* in (7a)) is promoted to subject but the rest of the underlying verb's complement frame (the direct object *the painting* in (7a)) is unaffected, deriving (7b). Adjectival participles, though, do not license the ‘secondary’ object *the painting*, as in (7c) shows.

**Table:** 

(7)	a.	Mary sold the collector the painting.
	b.	The collector was sold the painting.
	c.	*The collector is/seems/remains sold the painting.

It is relevant to the discussion that follows that adjectival participles may be classified as either ‘resultant state’ or ‘target state’ participles. Though these both display the properties of adjectival participles discussed above, resultant state adjectival participles such as *opened* imply the existence of an event falling under the underlying verb phrase denotation (here an opening event), while target state adjectival participles such as *closed* do not (Parsons 1990, Kratzer 1996, 2000, Embick 2004a,b and others). Intuitively, something that is closed might always have been closed, but something that is opened was necessarily opened by someone. Concretely, resultant state participles may not function as resultative secondary predicates, while target state participles may, as (8) shows. In this respect, target state participles pattern like ‘ordinary’ non‐derived adjectives such as *open*.

**Table:** 

(8)	Mary kicked the door *opened / open / closed.

Developments since Wasow's seminal work have shed substantial light on the internal workings of verbal and adjectival participles. I claim below, however, that contemporary approaches to adjectival passive participles fail to explain the ungrammaticality of the secondary object in (7c), and that the proper explanation for the ungrammaticality of (7c) points to the existence of a licensing requirement for DPs independent of Case. I present the background to this claim in the following section.

## 3 Word‐Internal Syntax

Wasow claims that verbal and adjectival participles are formed in different modules of the grammar. Adjectival participles are formed in the lexicon while verbal participles are formed in the syntax. However, more recent developments have shown that verb phrases are substantially more internally complex than Wasow envisioned, and these developments have provided alternative avenues for exploring the behavior of participles. These avenues have lead to the current conventional wisdom that both types of participles are formed in the syntax (Kratzer 2000, Anagnostopoulou 2001, 2003, Embick 2004a, Hallman 2013, Bruening 2014, Alexiadou, Gehrke and Schäfer 2014, Alexiadou, Anagnostopoulou and Schäfer 2015 and others), as described in detail below.

Bowers (1993), Hale and Keyser (1993), Collins and Thráinsson (1996), Marantz (1997), Chomsky (1995), Harley (1995), Kratzer (1996), Pylkkänen (2002) Travis (2010), and others (see Harley 2012 for an overview) claim that agentive verbs consist of a layer of structure that theta‐licenses an agent and that embeds a lower layer that theta‐licenses a theme. Following Chomsky (1995), I refer to the agent‐licensing higher layer as little‐vP and the lower theme‐licensing layer as big‐VP. On this view, a simple transitive construction like *Mary painted the door* has the theta‐structure in (9). A common semantic composition attributed to this structure relevant to the discussion below associates the eventiveness of *paint* (the action Mary undertook) with little‐v, and the result of this action (the door having a new coat of paint) with VP.

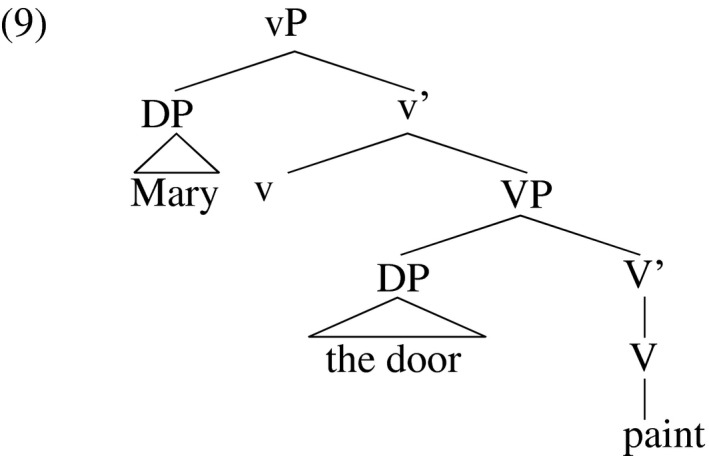

Implementing the idea that objective Case is contingent on the presence of an external argument (Burzio 1986), Chomsky (1995) and others postulate that vP Case‐licenses an object just when it theta‐licenses an overt agent. Chomsky (2000, 2001, 2004) claims that little‐v assigns Case through the ‘Agree’ relation, which extends from the ‘probe’ little‐v to the ‘goal’, the first accessible DP in its c‐command domain. Little‐v may act as a probe just when it also licenses an overt DP in its specifier position, the external argument. That external argument itself receives Case through an Agree relation with a finite T[ense] head. This situation is illustrated in (10). The Agree relation may trigger movement of the goal to the probe on a language‐specific basis, not shown in the tree below. The yet‐unoccupied subject position is marked [e] for ‘empty’.

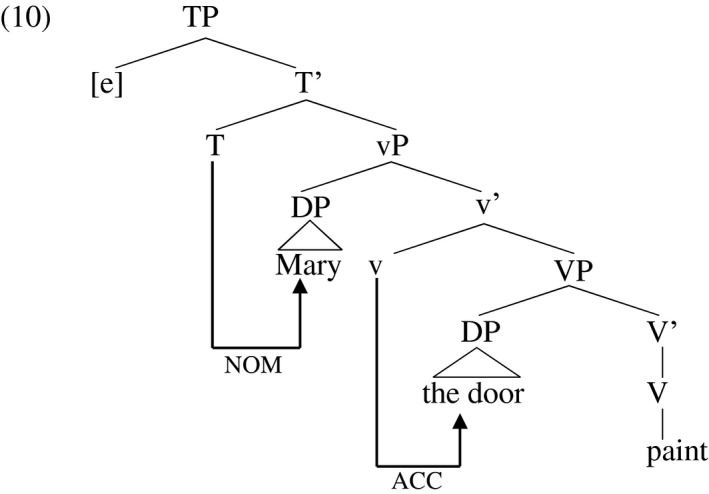

Embick (2004b), Folli and Harley (2005), Kallulli (2007) and others claim in slightly different guises that when little‐v is specified passive, it fails to act as a probe, and so does not license accusative Case on the object. The withdrawal of Case goes hand in hand with the ‘suppression’ of the external argument. A suppressed external argument may surface in a *by‐*phrase. I follow Bruening (2013) in analyzing the *by*‐phrase as an adjunct of v’ which saturates the theta role that v assigns. That is, it bears the same thematic relation to v as a ‘direct’ (i.e. bare DP) argument, but surfaces as an optional adjunct. Unable to receive Case from little‐v in this context, the object instead receives nominative Case from T. The agent is not accessible to the Agree relation extending from T since it is in the domain of a closer governor, the preposition. This situation is illustrated in (11). The usual position of the agent is marked [e].

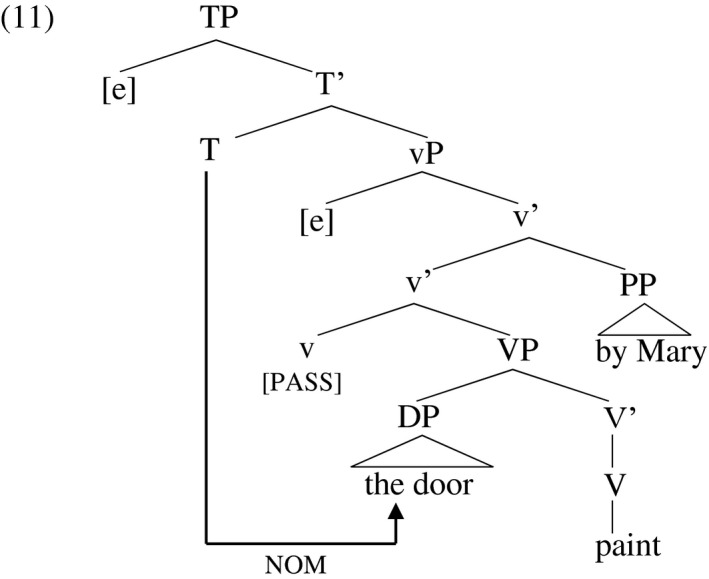

With this background, Embick (2004a) claims that adjectival passive participles are formed in the syntax, not the lexicon as Wasow claims, and that the difference between adjectival and verbal participles resides in how much syntactic structure is included within the participle. Evidence for the formation of adjectival participles in the syntax includes the fact that an adjectival passive participle may host a resultative secondary predicate like *flat* and *open* in (12a) and (12b) respectively. The main verbs *remain* and *look* demand the adjectival interpretation of the participles in (12) (Embick's example (74), p. 389). But the idea that the term *hammered flat* is formed in the lexicon is at odds with the notion of the lexicon as a word building module, while syntax is concerned with complex phrases. As complex phrases, *hammered flat* and *kicked open* must be built in the syntax.

**Table:** 

(12)	a.	The metal remained hammered flat.
	b.	This door looks kicked open.

Embick theorizes, then, that the difference between verbal and adjectival passive participles can be traced to the presence vs. absence of the little‐vP layer in their syntactic structure. In verbal participles, the participle is formed by applying the participle‐forming morpheme *–en* over the entire vP, so that the agent is still syntactically present within the participle (albeit as a *by‐*phrase or empty category, since the structure is passive). From the perspective of the idea outlined above that the eventiveness of an eventive verb is localized in vP, the inclusion of vP within the verbal passive participle ensures that the eventiveness of the underlying verb is preserved in the verbal participle.

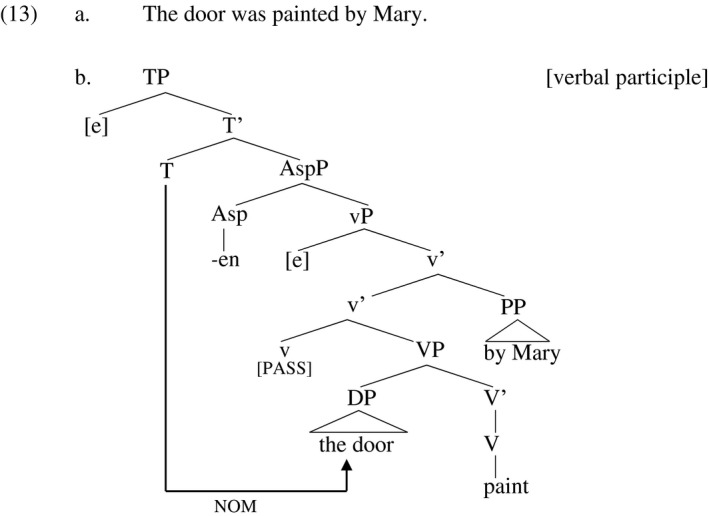

In the adjectival participle, on the other hand, Embick claims that the participle‐forming affix *‐en* applies at a lower level of structure, the level represented by big‐VP in the diagrams above, as illustrated in (14). This essentially strips away the agent‐ and event‐introducing little‐vP.

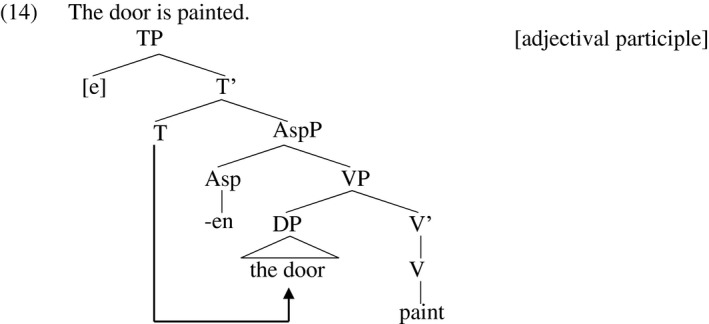

Embick decomposes what I label ‘VP’ into what he calls a ‘fientive’ little‐vP, responsible for telicity in adjectival participles, and ‘RootP’ designating the result state of the telic process. On this view, ‘target state’ participles, which are truly de‐agentivized adjectival participles, are formed from the bare RootP. Target state participles are not relevant for the argument about DP‐licensing that I make in what follows, so I merely note here that the analysis I propose below is compatible with the decomposition of VP into additional components along the lines of Embick (2004a).

In the structure in (14), the vP which is normally responsible for agentivity, eventiveness, and accusativity is missing, meaning the structure in (14) admits neither an agent (even in a *by*‐phrase), nor an accusative object, whose absence characterizes adjectival passives, as discussed in section 2. Further, the idea that vP is present in verbal passives but absent in adjectival passives predicts that verbal passives lack a ‘reflexive’ reading in examples like (6a), where the children may not be washing themselves. Baker, Johnson and Roberts (1989) claim that verbal passives contain a representation of the agent in the form of a pronoun, which I represent as a silent *pro* in (15a). When the object moves to subject position in verbal passives, it crosses over this pronoun. A pronoun crossed over in this fashion may in general not be referentially co‐indexed with the term that crosses over it, as the clearer case in (15b) shows, which does not admit a reading synonymous with *Who loves himself?* (Postal 1971, Wasow 1972). Postal and others call the impossibility of the co‐indexed reading in (15b) a ‘crossover effect’. Along the same lines, the pronoun representing the agent in the passive (15a) cannot be interpreted as co‐referential with the derived subject because that co‐indexation represents a crossover effect.

**Table:** 

(15)	a.	The children_i_ were being [_vP_ pro_*i,j_ washed *t*_i_ ]
	b.	Who_i_ does he_*i,j_ love *t*_i_

The fact that this cross‐over effect apparently does not arise in adjectival passives, then, supports the idea that vP is not present there, so there is no representation of the agent that could intervene in the promotion of object to subject.

And most importantly for the present purposes, the absence of vP in adjectival passives predicts the absence of accusative Case there, so that the object receives nominative Case from T instead, as in verbal passives (where accusative is also missing, but not because vP is missing altogether, but rather because passive vP does not license accusative). On this view, adjectival participles are structurally defective vis a vis verbal participles, and it is this structural defectiveness that accounts for the properties that distinguish them from verbal passives.

Although the view that vP is missing in adjectival participles therefore appears to have broad empirical coverage, in the following section I discuss developments since Embick's proposal that conflict with this characterization of adjectival passives, and that are particularly problematic in the face of the ungrammaticality of the ditransitive participle in (7c). I then propose an alternative analysis. This alternative is also based on the idea that adjectival participles are structurally defective, but maintains that it is D‐licensing structure that is missing from adjectival participles, not agent‐ and Case‐licensing structure.

## 4 Agentivity and Secondary Object Case in Adjectival Participles

Passivization in double object constructions in English leads to promotion of the indirect object to subject, as illustrated in (16).

**Table:** 

(16)	a.	The collector was sold the painting.
	b.	Mary was lent the car.
	c.	The participants were sent the program.
	d.	The best candidate was awarded the prize.

The examples in (16) show that the withdrawal of Case from the indirect object (*the collector* in (16a)), which triggers promotion to subject, does not affect the direct object (*the painting* in (16a)). Marantz (1993), Collins (1997), McGinnis (1998), Bruening (2001), Anagnostopoulou (2003), Woolford (2006) and others argue that indirect objects are introduced in the specifier of an ‘applicative’ head Appl, which occurs between agentive little‐vP and the underlying big‐VP. The base predicate structure for *Mary sold the collector the painting* looks like (17) on this view.

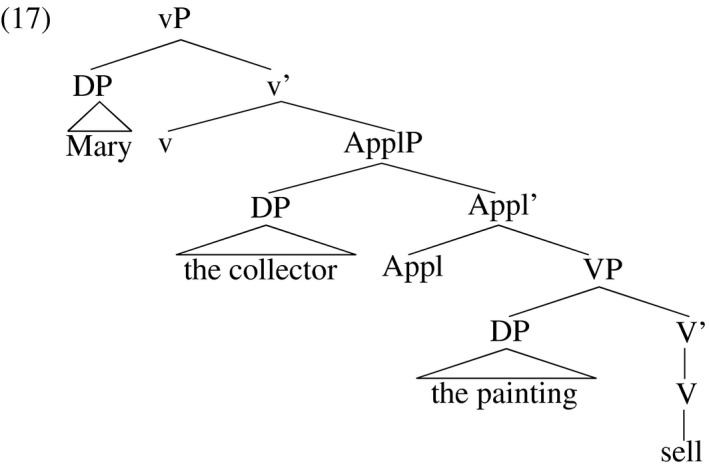

Ura (1996), Collins (1997), McGinnis (1998), Hallman (2015) and others claim that the direct object in (17) receives structural accusative Case from Appl while the indirect object receives structural accusative Case from little‐v. The analysis of passivization that relates it to the withdrawal of Case in vP predicts the pattern seen in verbal participles in (16)—the indirect object receives nominative Case from T instead of accusative from vP (and moves to subject position) and the direct object stays in the verb phrase where it continues to receive accusative Case from Appl. The withdrawal of the Case licensing potential of vP in no way affects ApplP, meaning passivization does not inhibit Case assignment to the direct object. The structure of (16a) is sketched in (18).

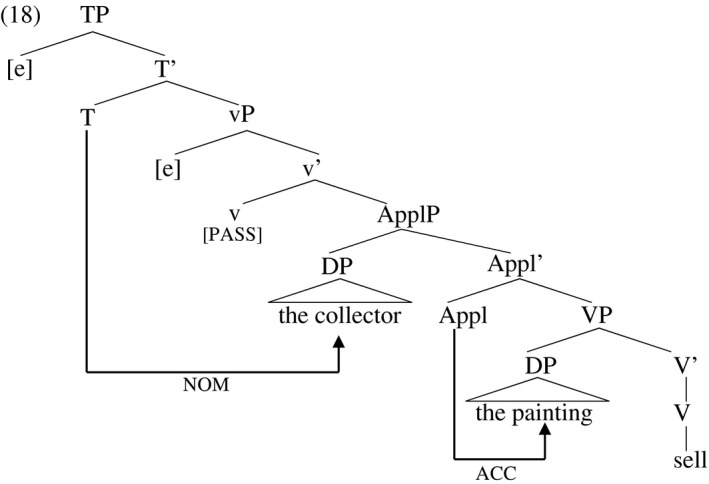

But just as the withdrawal of accusative Case at the level of vP in the verbal passive does not affect the licensing of secondary accusative on the direct object in (16), the ‘pruning’ of vP that Embick and others propose in adjectival passives is also not expected to affect licensing of the direct object in the adjectival passive counterparts to (16), which, as mentioned previously, are systematically ungrammatical, illustrated in (7c) above and in (19).

**Table:** 

(19)	a.	*The collector is sold the painting.
	b.	*Mary is lent the car.
	c.	*The participants are sent the program.
	d.	*Mary is awarded the prize.

Nothing discussed so far prevents ApplP from functioning as a base for the derivation of an adjectival participle, meaning it is unclear at this point what rules out the examples in (19). In the following section I argue against a plausible null hypothesis on the matter, and then propose an alternative.

## 5 How Big are Adjectival Participles?

A natural explanation for the ungrammaticality of (19) is that not only vP is missing from adjectival participles, but ApplP as well. That is, the participle‐deriving operation may either apply to vP, deriving a verbal passive participle, or to VP, deriving an adjectival passive participle, in the context of which neither an agent nor a recipient (indirect object) argument may surface (Anagnostopoulou 2006 describes an analysis of this kind for nominalizations). In this section, I argue against this view, reviewing some recent developments in the analysis of participles that lead to the conclusion that at least some adjectival participles in fact *do* contain vP. Those participles that contain vP are expected to be able to contain ApplP as well on the assumption that syntax is monotonic—the presence of higher structure implicates the presence of lower structure. This consideration undermines the potential analysis that takes adjectival participles to be formed over VP and to exclude vP and ApplP, and in turn necessitates a different explanation for the ungrammaticality of the examples in (19).

It turns out that the impossibility of *by‐*phrases and other agent‐oriented material in resultant state adjectival passives has been overstated. Agent‐oriented material is not excluded in principle, but is *restricted* in adjectival passives for reasons discussed in detail by Rapp (1997), Maienborn (2007, 2009), Meltzer‐Asscher (2011), Gehrke (2011, 2015), McIntyre (2013, 2015), Bruening (2014), Alexiadou, Anagnostopoulou and Schäfer (2015), and others.

For example, while the examples in (20) are judged ungrammatical in the literature, Maienborn points out that (21a) is natural, and Gehrke that the example in (21b) is natural.

**Table:** 

(20)	a.	*This manuscript is cited by John
	b.	*This picture looks drawn by John.

**Table:** 

(21)	a.	This manuscript is cited by Chomsky.
	b.	This picture looks drawn by a child.

Maienborn claims that adjectival participles denote ‘ad‐hoc’ properties related only pragmatically to the compositional semantic content of the participle. What one means by (21a) is that the manuscript is significant, having been cited by no less a figure than Chomsky, and by (21b) that the drawing is sloppy. Here, ‘significant’ and ‘sloppy’ are the properties that these participles refer to ad‐hoc. This effect fails in (20) because no prominent personality comes to mind known simply as ‘John’, leaving us no clues about what property might be being referred to ad‐hoc in this case. Extending this view, Gehrke claims that the underlying verb in adjectival participles like *cited by Chomsky* describes, in contrast to the corresponding verbal participle, an event *kind*, rather than an event *realization* (a specific event instantiating the kind; see Carlson 1977 on the kind/realization contrast). The state they describe, then, is not simply one resulting from a particular being‐cited‐by‐Chomsky event, but rather one that would have resulted from *any* typical being‐cited‐by‐Chomsky event. This view extends naturally to the contrast between (20b) and (21b), where the indefinite *a child* more readily supports an event kind description than the proper name *John*, since pictures drawn by children have consistent identifying features. (21b) says that the picture in question has features that any typical being‐drawn‐by‐a‐child event would impart to it. This view correctly predicts that indefinites will generally be better than proper names in *by‐*phrases in adjectival passives, since indefinites contain a description, whereas names refer directly. Adjectival participles must, however, contain vP so that *by‐*phrases are licensed at all. The literature cited above presents an explanation for the marginality of many cases of adjectival participles with *by‐*phrases that is compatible with the general possibility of having vP within the adjective. In light of this, it appears that *by‐*phrases are not excluded structurally from adjectival passives, but that they are militated against by semantic compositional factors.

Just as the apparent exclusion of *by*‐phrases from adjectival participles has a potential independent explanation other than the wholesale absence of vP, McIntyre (2013, 2015), Bruening (2014), Doron (2014), Alexiadou, Anagnostopoulou and Schäfer (2015) and others claim that the cross‐over effects discussed in section 2 are less robustly associated with adjectival participles than previously believed. They cite attested examples of adjectival participles that show disjoint reference effects of the kind claimed to arise from the cross‐over restriction. For example, children who for some reason frequently punish themselves cannot be described as *frequently punished children*, and a door that opened by itself cannot be described as *an opened door* (Bruening 2014, p. 382). If cross‐over is the correct explanation for obligatory disjoint‐reference between the derived subject and the implicit agent, then these observations indicate that these adjectival participles contain a representation of the agent that intervenes in the promotion of object to subject. McIntyre notes that English adjectival participles that do not display cross‐over effects usually involve verbs that are in some sense ‘naturally’ reflexive, such as *wash*. But outside of this class, vP and its associated agent appears to not be excluded from adjectival participles in principle.

These facts support a syntactic analysis of adjectival participle formation in general, since externalization potentially shows configurationally determined cross‐over effects. And they show that it is in principle possible for adjectival participles to be formed from a structure that contains a syntactic representation of the agent, even when this argument is not overt in passive participial structures. Since ApplP occurs between vP and the verbal root in VP in ditransitive constructions, a participle that includes vP is large enough to include ApplP.

This conclusion is based on the premise that syntactic structure is monotonic in the sense that the presence of a certain category (e.g. vP) implies the presence of structure hierarchically below it but not structure hierarchically above it. Of course, the structure in question may be subject to additional selectional requirements. ApplP only occurs when the underlying verb has a recipient or beneficiary argument, as in the case of *give*. Consequently, the conclusion that vP may occur in adjectival participles does not mean that adjectival participles *necessarily* contain ApplP. Only ditransitive verbs project ApplP in the first place. But if an adjectival participle of a ditransitive verb contains vP, then, by the premise that syntactic structure is monotonic, ApplP is present in the adjectival participle as well. The inverse does not hold. An adjectival participle that contains ApplP might not contain vP according to the monotonicity premise. A participle might also include neither vP nor ApplP. This characterizes the target state participles discussed briefly in section 2; they describe a state with no event or agentivity implications. But the fact that vP and ApplP are not excluded in principle from adjectival participles is difficult to reconcile with the facts in (19), where a direct object is excluded from an adjectival participle for which a result state reading is available. As Wasow shows, the offending feature of (19) is the occurrence of the direct object there. If vP, and therefore ApplP, may occur in adjectival participles, the ungrammaticality of (19) remains puzzling.

Pesetsky (1995) presents an analysis of facts similar to those at issue here that offers another possible explanation for the impossibility of indirect objects in adjectival passives. As Kayne (1984, p. 156) demonstrates, nominalizations of double object constructions are not possible, as seen in (22a). Nominalization of *give* is possible in the prepositional frame in (22b); it appears to be the double object structure that undermines the nominalization, parallel to adjectival passives.

**Table:** 

(22)	a.	*Sue's gift of Mary of a book.
	b.	Sue's gift of a book to Mary.

Pesetsky claims that the double object construction contains a covert preposition *G*, as shown in (23), which assigns Case to the theme and subsequently raises and affixes to the main verb. As such, *G* is parallel to Appl in the present analysis (except that Appl in turn embeds VP on this account). The resulting verbal head complex consists syntactically of the verbal head V and the incorporated covert preposition *G*. Pesetsky then claims that a word suffixed by *G* cannot undergo further affixation. This blocks both nominalizations derived from double object constructions like (22a) and the corresponding adjectival passive participles like those in (19). He claims that in the prepositional frame that forms the derivational base for (22b), *G* surfaces in the form of the overt preposition *to*, and does not incorporate into the verb and so does not prevent further derivation to an adjective.

**Table:** 

(23)	a.	[_V_ give Mary [_PP_ *G* a book ]]	→
			[_V_ give+*G*_i_ Mary [_PP_ *t*_i_ a book ]]
	b.	[_V_ give a book [_PP_ to Mary ]]	

Pesetsky (pp. 81–93) proposes that *G* belongs to a set of affixes that block most other affixes, mimicking attested morphological selectional restrictions in English (Fabb 1988). But this view is in conflict with the fact that in the structure posited, *G* is not actually an affix of the higher V head, but vice versa. The adjectivizing morpheme (whether *‐en/‐ed* itself or a covert derivational morpheme) applies outside VP on Pesetsky's analysis. Head movement of V, with *G* adjoined to it, to the adjectivizing head creates a head complex [[[*G*]_P_ +V]_V_ +A] _A_. It is V that is structurally adjacent to the adjectivizing head in this structure, not *G*. The condition that blocks the derivation of an adjective (or a noun) in the presence of *G* is not a condition on what the adjectivizing head A directly attaches to but rather on what may attach to the head that A in turn attaches to. This is an unusual, non‐local interaction between the adjectivizing morpheme and something contained in its complement.

I proceed in the following section to present an alternative analysis of the ungrammaticality of the data in (19) based on D‐licensing, which captures the effect in (19) in terms of a minor modification of the standard view that adjectival participles are structurally defective, but yet is compatible with evidence that adjectival passives contain entire vPs, which may include ApplP.

## 6 D‐Feature Checking in Verbal and Adjectival Passives

I conclude from the discussion above that adjectival participles may be derived over vP and nothing blocks ApplP from occurring in such participles. If ApplP occurs, it should not only introduce an indirect object but also assign Case to the direct object in contexts like (19). The fact that the examples in (19) are ungrammatical, then, means that their ungrammaticality is not reducible to a failure of Case licensing.

I propose therefore that Case is not the only licensing requirement on DPs, but that DPs bear another feature that requires syntactic licensing for the derivation containing it to converge. There is some precedent for the notion that DPs bear a ‘categorial’ feature, that is, a feature reflecting their syntactic categorial status as DPs. Chomsky (1995) introduces the notion as an unsaturated feature of the subject position in English, which is saturated by movement of a constituent with the categorial feature ‘D’ to it, motivating insertion of expletive subjects when no subject is available. This notion of D‐feature checking subsumes the Extended Projection Principle (EPP), which requires every sentence to have a subject. Here, I propose a notion of D‐feature checking that makes the checking requirement a requirement of DPs, like Case is, so that a DP that fails to occur in a D‐feature checking position at some point in the derivation will prevent the derivation from converging. I propose that the EPP is a specific attribute of the highest D‐feature licensing position to the effect that unlike other D‐licensing positions, it not only *may* license a D‐feature but *must*, and this requirement goes hand in hand with movement of the targeted DP to the D‐licenser in English. I expand on this matter in sections 7, 8 and 9.

The proposal that DPs are subject to a D‐licensing requirement in addition to their Case requirement allows us to explain the ungrammaticality of (19) in terms similar to Embick's (2004a) paucity‐of‐structure view of the properties of adjectival participles. Adjectival participles do not contain enough structure to license a DP object. But the missing structure is not Case‐licensing structure, but D‐licensing structure. I suggest that D‐licensing for internal arguments emanates from dedicated syntactic heads that lie outside of vP, that I label ‘Δ’, and the corresponding projection ΔP ‘Delta Phrase’.

The verb phrase structure for the active double object construction in (24a) looks like (24b) on this proposal. Case‐ and D‐licensing of the agent *Mary* by T and an adjacent Δ higher up in the structure is not shown. Just as accusative Case extends from the probes v and Appl to the goals in ApplP and VP respectively, D‐feature licensing extends from the two Δ probes above vP to the respective DPs. Both Case‐ and D‐licensing may in principle involve raising of the DP to a projection of the probe, which may be optional or obligatory depending on the parametric specification of the language. Movement is not shown here. Since I will sometimes want to refer to the individual ΔP projections specifically, I number them consistently in the trees to follow, beginning with the highest (not shown in (24)).

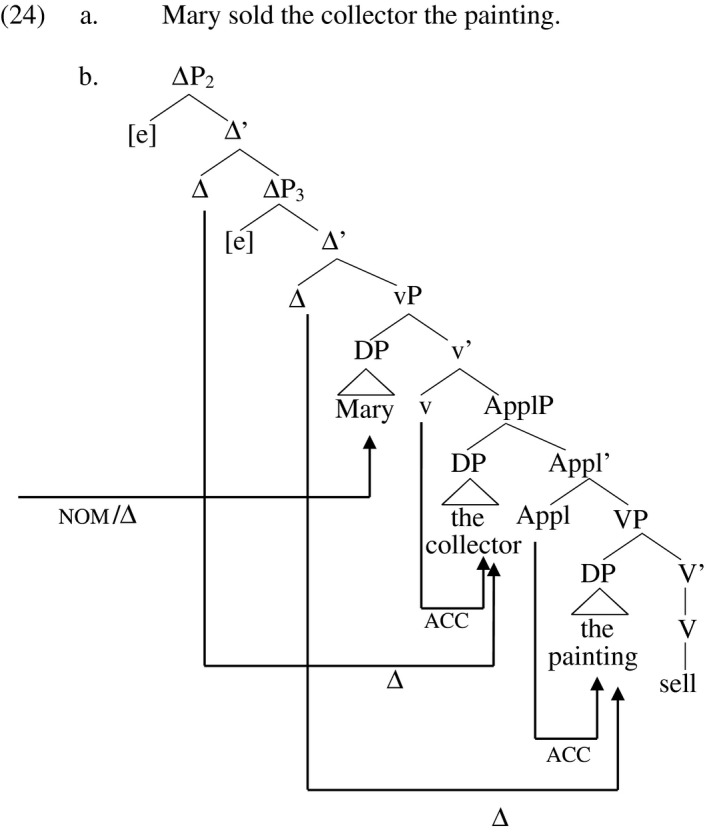

In the tree in (24b), the highest Δ head (of ΔP_1_, not shown) licenses the highest DP in the predicate, the next highest Δ head the next highest DP, and the lowest Δ head the lowest DP. This alignment is not critical for this particular example but it is critical in certain other contexts that I describe later. A Δ head therefore does not necessarily D‐license the closest DP. Rather, D‐feature licensing belongs to a class of phenomena that are subject to a parallelism, or homomorphism, requirement between the derived and the base order, such as wh‐movement in Slavic languages (Rudin 1988, Richards 1997), quantifier raising in English double object constructions (Larson 1990, Bruening 2001), and ordering restrictions between verbs, adverbs, and objects in Scandinavian languages (Holberg 1986, Fox and Pesetsky 2005). These phenomena all involve terms whose hierarchical order in the base structure must be preserved in the derived structure. Various mechanisms have been proposed to accommodate such phenomena, including a version of the ‘Shortest Move’ principle stating that if the lower of two licensers were to license the higher of two licensees, then the licensing relationship between the higher licenser and the lower licensee would be too long, necessitating the homomorphic order instead (Chomsky 1995). Richards (1997) and Bruening (2001) postulate a mechanism that results in the second of two licensees ‘tucking in’ under the derived position of the first, while Fox and Pesetsky (2005) postulate a principle that requires hierarchical relations established in one phase to be preserved in higher phrases. Though the issue of which of these views best captures the phenomenon might warrant further investigation, for concreteness I adopt Fox and Pesetsky's view here, in conjunction with the premise that vP and PP are phases. Consequently, D‐licensing must match the base order of arguments in vP, but if an argument occurs in a PP, including *by*‐phrases, it is not ordered with respect to other arguments in the vP phase. After Fox and Pesetsky, I call this the ‘Cyclic Linearization’ requirement.

Before proceeding, I address another aspect of the licensing relations depicted in (24b). I have claimed that DPs need to be D‐licensed, but not that Δ heads necessarily have to license a DP. Rather, Δ heads are specified individually for whether they probe obligatorily or optionally. The highest Δ head probes obligatorily in English, yielding the effect of the EPP, but the other, lower Δ heads probe optionally. A Δ head specified to probe optionally that does not license a DP does not in any way undermine a derivation. But a DP that is not licensed necessarily undermines a derivation. Where probing is optional, no grammatical mechanism guarantees that DPs will be D‐licensed. In this respect, D‐licensing is like Case licensing. No grammatical force requires Case licensing heads to probe for a goal, but if any DPs fail to be assigned Case, the structure is ruled out by the Case Filter. The Case Filter itself is subsumed in Chomsky 1995 by a generalized condition requiring derivations to ‘converge’ at the interfaces, which they do when they contain no unlicensed features. The only structures that survive at the interfaces, then, are those in which each DP stands in an Agree relation with some Δ head, there is no general requirement that each Δ head stand in an Agree relation with some DP (though a specific requirement to this effect might hold for some Δ heads). In (24b), every Δ head stands in the Agree relation with some DP, but this is not the case in the passive, as described below.

In the verbal passive, shown in (25), little‐v is marked as passive, with the effect that the argument it projects (agent) occurs in a *by‐*phrase adjunct of v’ (or is altogether covert) and little‐v no longer assigns Case. I set aside many questions about the relation between passivization and its morphological expression and assume that the presence of *‐en* on the participle is conditioned by passivization in some fashion. The occurrence of *‐en* in turn prevents the verb from hosting finiteness morphology and necessitates insertion of the auxiliary *be* for this purpose. Just as the preposition *by* checks the Case of the agent in the passive, I assume that a ΔP is available within the *by*‐phrase as well, that checks the agent's D‐feature, a point I expand on below. As a result, the argument that receives accusative from little‐v in the active (the recipient) receives nominative from T in the passive, while the theme receives Case as before from Appl. In principle, the three ΔPs found in the active in (24) are available in the verbal passive in (25) (ΔP_1_ being adjacent to TP and not shown in the tree). Only two DPs remain to be licensed in the passive though, since the agent is licensed within the *by*‐phrase (when overt at all). This is compatible with the view sketched above that D‐licensing is a requirement of DPs, not ΔPs. DPs are licensed under Agree with Δ, but whether a given Δ probes or not is specified microparametrically; it is not a grammatical necessity. The important thing is that there are at least as many ΔPs in the structure as DPs to be licensed. A Δ that does not itself probe is syntactically inert and does not intervene in the Agree relation between a higher Δ and a lower DP. The superfluous non‐probing Δ_3_ in the tree in (25) is grey‐out to indicate its syntactic inertness.

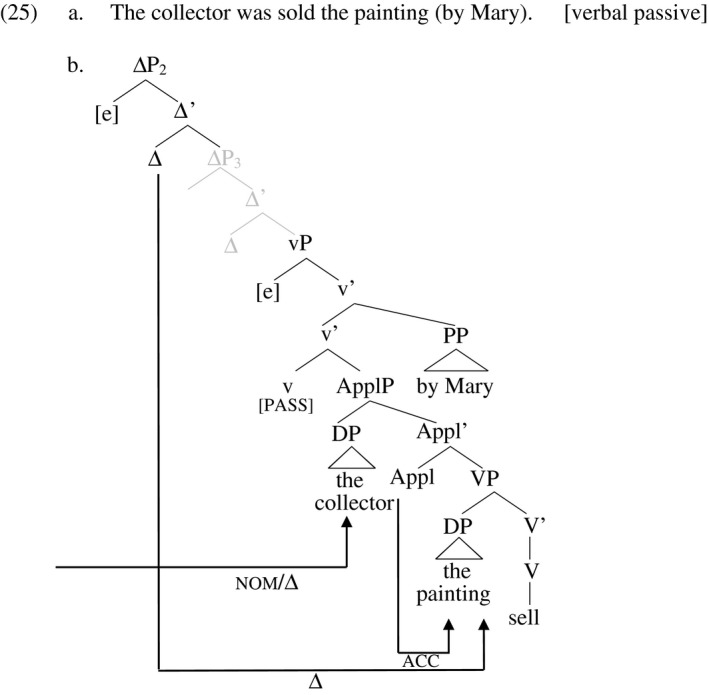

Note that it does not matter whether the remaining object DP is licensed by ΔP_2_ or ΔP_3_. In the diagram, ΔP_3_ is inert but could license the theme if ΔP_2_ were inert instead. I continue to assume that D‐licensing chains are subject to Cyclic Linearization; the highest available Δ licenses the highest available DP, the next highest the next highest, etc. Further, the EPP remains in effect, here in the form of the requirement that ΔP_1_ (above TP, not shown), both obligatorily probes for and attracts a DP goal. That is, ΔP_1_ is never inert in English, and the DP it attracts must be the highest available DP due to the Cyclic Linearization requirement. I assume that the *by‐*phrase PP is a barrier to the Agree relation, meaning the DP internal to the PP is not visible to the ΔPs external to it. Rather, the DP internal to the PP is licensed by Case and D‐licensing projections also internal to the PP, so that the PP has an internal structure similar to that of verb phrases, as sketched in (26). Here, little‐p assigns Case to the object of big‐P and Δ licenses its D‐feature, both under Agree. Movement of big‐P to little‐p derives the order preposition > object.

**Table:** 

(26)	[_ΔP_ Δ [_pP_ p [_PP_ Mary by_P_ ]]]

In this analysis, D‐licensing is disconnected from Case‐licensing. The feature that modulates object Case assignment, namely the [pass] feature on little‐v, does not affect the availability of D‐licensing positions above the verb phrase. I propose that what does affect the availability of D‐licensing positions, though, is the process that forms adjectival passive participles. This is because, as Embick (2004a) claims, adjectival participles are ‘truncated’ with respect to the corresponding verbal structure. Specifically, I propose that the predicate‐level ΔP positions shown in the trees above (ΔP_2_ and ΔP_3_) are not available in adjectival passives, though the higher ΔP_1_ adjacent to TP (not shown) remains available for the subject (neither verbal nor adjectival passivization affects the availability of nominative Case or D‐licensing for the subject, though it affects which argument is promoted to subject). That is, the adjectival participle is formed over vP, and no additional object licensing structure is projected in adjectival passives. This is a version of Embick's analysis, in which adjectival participles are smaller than verbal participles. Embick claims that adjectival participles lack vP (and ApplP), but the evidence reviewed in section 5 indicates that vP and ApplP are present in adjectival participles. The idea that adjectival participles lack ΔP is a way of reconciling evidence for vP and ApplP in adjectival participles with the idea that adjectival participles are reduced, or defective, verb phrases. They are defective in that they lack ΔP.

I notate the operator that derives adjectival participles as ‘A’ and its maximal projection ‘AP’. The tree in (27) represents the structure of the adjectival participial derivative of (25b), in which the *by‐*phrase is omitted; the agent is represented as the empty category [e]. This AP occurs under a copular auxiliary *be* (not shown), which verbalizes the structure and itself moves to the tense node T (not shown). What makes (27a) ungrammatical is that although the direct object has its Case licensed by Appl, there is no structure available to license its D‐feature. A reviewer of the present work suggests that the absence of D‐licensing structure in adjectival passives may result from selectional restrictions. If the auxiliary *be* selects either ΔP (in verbal passives) or AP (in adjectival passives), and A selects vP directly, not ΔP, then there is no way for ΔP to occur in the context of an adjectival predicate.

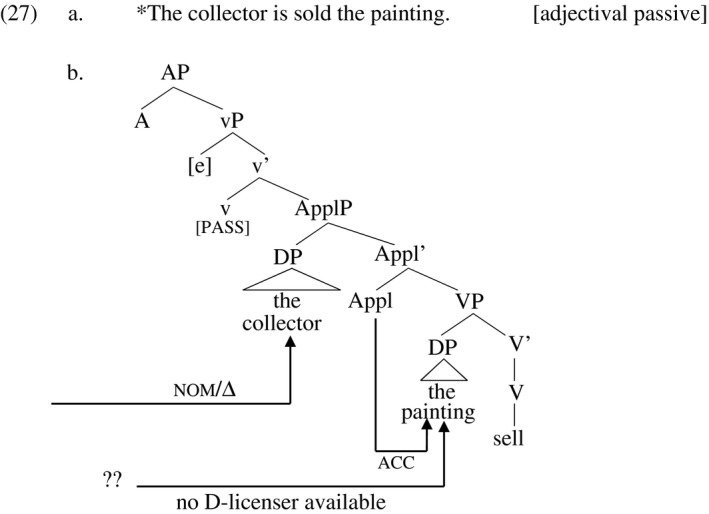

The overall effect of this analysis is that a theme may occur as object in verbal but not adjectival passive participles. As such, it presents part of an explanation for what was once construed as a thematic restriction on adjectival passives, to the effect that only the theme may occur as the external argument of an adjectival passive (Wasow 1980). In the present proposal, this is because the theme fails to be D‐licensed if the indirect object promotes to subject. This proposal also predicts, though, that it should be possible to externalize a non‐theme argument if the theme occurs in a prepositional phrase, since the preposition introduces Case and D‐licensing structure of its own. This is precisely the case in the *with*‐alternant of the spray/load alternation, illustrated in (28a). *The hay* is the theme in (28a)—it is what undergoes a change of location—while *the truck* designates the location. Yet, it is *the truck* that is externalized in the adjectival passive based on (28a), illustrated in (28b).

**Table:** 

(28)	a.	We loaded the truck with hay.
	b.	The truck remains loaded with hay.

On the present analysis, externalization of *the truck* is possible because *the hay* is both Case‐ and D‐licensed within the prepositional phrase *with the hay*, and so promotion of the location argument *the truck* to subject, where it is Case‐ and D‐licensed in the vicinity of tense, does not leave the theme with no possibility to be D‐licensed. The preposition D‐licenses the theme in (28b) even when no other D‐licensing structure is available.

This analysis mimics Levin and Rappaport's (1986) explanation for what they call the Sole Argument Generalization, which states that whenever an object may stand alone in the verbal construction, that argument may be externalized in the adjectival derivative. For example, the verb *stuff* patterns like *load* except that in the locative format illustrated in (29b), the location argument is obligatory, while in the *with*‐variant in (29a) the theme is optional. This is unlike *load*, where the location in the locative frame is optional (29c).

**Table:** 

(29)	a.	We stuffed the pillow (with feathers).
	b.	We stuffed the feathers *(in the pillow).
	c.	We loaded the hay (onto the truck).

The pattern in (29) carries over to the adjectival derivatives. In the *with*‐variant in (29a), the location argument *pillow* may be externalized, as shown in (30a) (just like *load* in (28b)). But in the locative variant in (29b), the location cannot be externalized unless the location argument is overtly expressed, as in (30b).

**Table:** 

(30)	a.	The pillow remains stuffed (with feathers).
	b.	The feathers remain stuffed *(in the pillow)

Thus, an internal argument can be externalized just when it can stand alone in the corresponding active. As Levin and Rappaport emphasize, (30b) represents the same fact as (29b). They state explicitly that these observations support the claim that adjectival passives are derived from verbal passives, as I claim here. Since *the feathers* cannot occur in the verb phrase without a location argument such as *in the pillow*, the adjectival derivative in which *the feathers* is externalized is also ungrammatical without the location *in the pillow*. What remains puzzling is the fact in (29b) and in particular the contrast to *load* in (29c). It is unclear why the location is obligatory in the locative frame for *stuff* but not in the corresponding example with *load*. The answer to this question, though, has nothing to do with the derivation of adjectival participles, it is a fact about the argument structures of the verbs involved. Levin and Rappaport stipulate the difference between *stuff* and *load* in their respective lexical entries as superficial lexical idiosyncracies.

As Levin and Rappaport claim, the Sole Argument Generalization is itself an epiphenomenon of the fact that the expression of some arguments is contingent on the expression of others and that an argument in a prepositional phrase is unaffected by adjectivization. Levin and Rappaport claim that the effect of adjectivization is the withdrawal of Case. The present study attempts to show that for structural reasons, there is no reason to believe that secondary object Case is withdrawn in adjectival passives, and that another explanation is necessary, which I claim is D‐licensing.

This analysis applies the hypothesis that adjectival participles are smaller than verbal participles in a new way. They are smaller, but they contain vP and may in principle host agent‐oriented modificational material, modulo restrictions related to the aspectual profile of adjectival participles discussed in section 5. What they lack is D‐licensing structure. In the following section, I show that facts from German support this analysis.

## 7 Adjectival Participles and Inherent Case in German

In this section, I argue further against any Case‐theoretic analysis of the ungrammaticality of sentences like (19) and in favor of the idea that D‐licensing is the critical factor. The facts come from German, where double object constructions display a rather different Case frame than in English. In spite of the difference in Case frames, the two languages show exactly the same restrictions on object licensing in adjectival participle constructions, restrictions that I claim are reducible to the failure of D‐licensing in the relevant cases.

In German, the recipient argument in transfer‐of‐possession double object constructions receives dative Case, and the theme receives accusative, as shown in (31). I follow the practice in German linguistics of citing examples in the form of subordinate clauses so that observations about word order are not confounded by topicalization, which is only available in root environments.

**Table:** 

(31)	weil	Maria	dem	Sammler	das	Gemälde	verkauft	hat.
	because	Maria	the_DAT_	collector	the_ACC_	painting	sold	has
	‘because Maria sold the collector the painting.’							

Dative is an ‘inherent’ Case, meaning it cannot be withdrawn in the course of a derivation, for example through passivization. When (31) is passivized, the direct object receives nominative Case and controls agreement on the verb, but the indirect object remains dative, as shown in (32) (coincidentally nominative and accusative neutralize morphologically for the singular neuter noun *Gemälde* (painting), but the two Cases are paradigmatically distinct). The canonical word order, however, is parallel to English. Although German displays more flexibility in word order than English, the dative recipient precedes the nominative theme in the order that is judged to be unmarked in the passive. Note that German uses the dedicated auxiliary *werden* ‘become’ for the verbal passive, distinct from the copular auxiliary *sein* ‘be’.

**Table:** 

(32)	weil	dem	Sammler	das	Gemälde	verkauft	wurde.
	because	the_DAT_	collector	the_NOM_	painting	sold	became
	‘The collector was sold the painting.’						

Czepluch (1996) and Wunderlich (1996) claim that dative Case on recipients in German is assigned configurationally in an extended projection of VP, where the recipient is base generated. That is, Case assignment is configurational, but targets the base position of the recipient, hence its ‘inherent’ association with that theta role. McFadden (2006) claims specifically that a recipient is dative in German in virtue of occurring in the specifier of ApplP (its base position). McIntyre (2006) and Meinunger (2006) adopt this same view for German, though McIntyre calls the projection in question V^DAT^P and Meinunger ‘Poss[ession]P’; McGinnis (1998), Anagnostopoulou (2003), Woolford (2006) and others make this same claim for various other languages. Following this line, I claim specifically that dative is assigned in German by Appl to its specifier, the base position of the recipient, rather than under Agree to the lower theme. English and German differ, then, in the configuration under which Appl assigns Case. In English, it assigns Case to a DP it c‐commands. In German, it assigns Case to its own specifier, which is also a theta‐position. This means that the recipient enters the derivation Case‐licensed in German but not English.

The fact that the theme receives nominative in the passive indicates that the source of accusative Case for the theme in the active construction is little‐v, rather than Appl as in English, since passivization is reflected in the [pass] feature on v, associated with suppression of the agent (Embick 2004b, Folli and Harley 2005, Kallulli 2007). Assuming that D‐licensing proceeds as in English, the derivation of the active construction in (31) looks like (33).

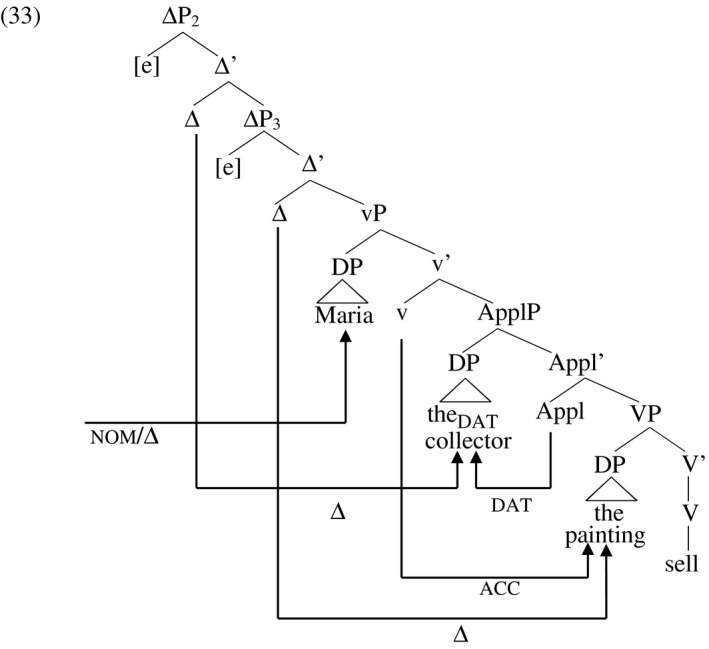

This tree resembles English in terms of D‐licensing but in terms of Case licensing, it differs from the corresponding English construction diagrammed in (24). In English, the higher Case assigner little‐v licenses Case on the higher of the two arguments (the recipient), and the lower Case assigner Appl licenses Case on the lower of the two (the theme). In German, the recipient receives Case in its base position. I assume this accounts for its lack of intervention in the Agree relation holding between little‐v and the theme in German, which crosses over the dative recipient. Having been Case‐licensed in its base position, the dative recipient is rendered inactive and invisible to other Case chains, in the active as well as in the passive discussed below.

In the verbal passive construction in (32), accusative Case in vP is withdrawn, and the theme receives nominative Case from finite T while the recipient continues to receive dative from Appl in situ. The verb agrees with the theme (the nominative element) by virtue of the Agree relation between the theme and T. However, even though the theme receives Case from a higher Case assigner (T) than the recipient (Appl), the canonical word order reflects that found in English: the recipient precedes the theme in the order regarded by native speakers as unmarked. In the context of this analysis, this means that the unmarked order is the one that meets the Cyclic Linearization requirement on D‐licensing. The canonical linear order of arguments reflects not the hierarchical order of Case assigners but the hierarchical order of the Δ heads that D‐license the recipient and theme respectively. The unmarked order is merely a preference in informationally neutral contexts. All logically possible orderings of the three arguments in German ditransitives are grammatical word orders, but signify non‐neutral information packages (Diesing 1992, Pafel 2005). I propose that these word orders are derived by movement of arguments to their respective D‐licensor, but that Cyclic Linearization is merely the unmarked preference, deviations from it being allowed when these are accompanied by the intention to signal non‐neutral propositional content. In this language, then, D‐licensing is obligatory (as we will see below) but movement to the respective ΔP is optional, as is adherence to the Cyclic Linearization requirement. The tree for (32) is sketched in (34). The Δ_3_ head does not probe and is greyed‐out here as in the English example in (25). This choice is for the sake of concreteness; as in English, it does not matter which of Δ_2_ or Δ_3_ probes in this example.^3^

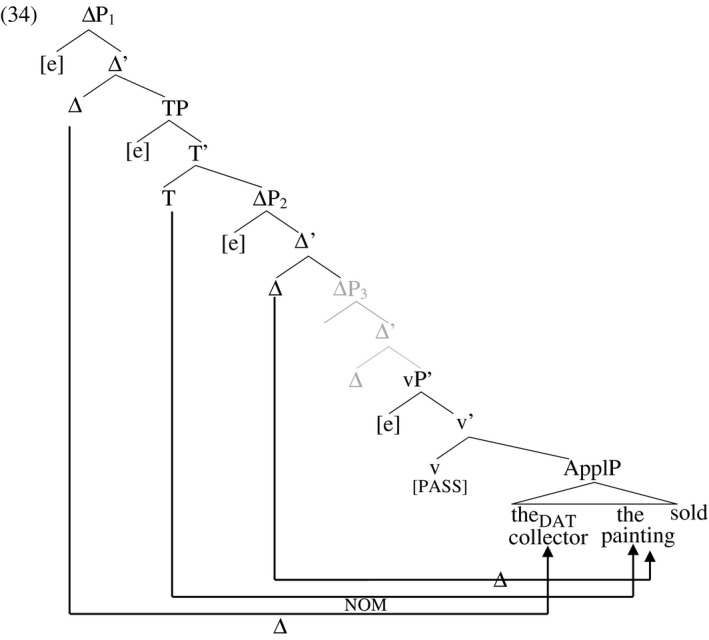

With this analysis of active and passive structures in German in hand, I now point out that the loss of ΔP_2_ and ΔP_3_ in adjectival participles is expected to have the same effect in German as in English, in spite of the different source for the Case of the direct object in German passives (TP instead of ApplP). Indeed, as in English, adjectival participles of double object verbs in German do not admit an internal direct argument, as shown in (35). As mentioned previously, the adjectival passive is morphologically explicitly marked in German with the auxiliary *sein* ‘be’, as opposed to *werden* ‘become’ that marks the verbal passive.

**Table:** 

(35)	*weil	dem	Sammler	das	Gemälde	verkauft	ist.
	because	the_DAT_	collector	the_NOM_	painting	sold	is
	*‘because the collector is sold the painting.’						

If Case is the only licensing requirement on DPs, the ungrammaticality of (35) is puzzling, albeit for a different reason than the ungrammaticality of the corresponding English examples in (7c)/(19). The English example (seen in the translation to (35)) is puzzling because whatever assigns Case to the direct object in the corresponding verbal passive in (2b) should still be present in the adjectival participle, given evidence that both vP and ApplP are present in the participle. So the ungrammaticality of the English example cannot follow from the unavailability of a Case position for the accusative direct object. The problem in German is even more evident: the verbal passive example shows both that the indirect object receives inherent Case, which should still be assigned in the participial derivative, since it is assigned in the base structure, and that the object may receive nominative Case from T even without raising to [spec,TP]. These ingredients are expected to make (35) possible, where the indirect object has dative Case and the direct object receives nominative. Adjectival passive formation is not expected to block either inherent Case assignment nor the assignment of nominative from T. Consequently, it is unclear what rules out (35) if the only ingredient of DP licensing is Case. The D‐licensing analysis proposed here derives the ungrammaticality of both the English examples in (19) and corresponding German examples such as (35) in spite of the different Case assignment configurations found in the two languages.

## 8 The Relation Between D‐licensing and Definiteness

D‐licensing was originally proposed in connection with expletive subject constructions in English and Icelandic in Chomsky 1995, as a refinement of the Extended Projection Principle (EPP), which requires every sentence to have an overt subject in English (Chomsky 1981, 1986). Chomsky claims that the tense head T bears a D‐feature that must be licensed by the occurrence of a DP in [spec,TP], motivating insertion of an expletive pronoun *it* (36a) or *there* (36b) if no other DP occurs in [spec,TP].

**Table:** 

(36)	a.	It is raining.
	b.	There arrived a man.

D‐licensing in this usage does not literally mean ‘definiteness’ licensing, since indefinite subjects satisfy the EPP in English, and even *it* and *there* in expletive subject constructions cannot be said to be semantically definite, since they are non‐referential. D‐licensing refers to a formal syntactic requirement that a constituent with the category DP be present in the subject position, even if indefinite or vacuous, not a requirement that the subject be definite. In what follows, though, I claim that it is possible for D‐licensing to be sensitive to definiteness, and that this view goes some way in explaining why some languages seem to lack a double object construction. Specifically, I claim that this proposal captures a definiteness effect on indirect objects in Maltese that, much like adjectival passives in German, evades a Case‐based analysis, supporting the general program fleshed out here on the independence of Case and D‐licensing.

Maltese is a peripheral dialect of Arabic spoken on the island of Malta and elsewhere. Double object constructions in Maltese are essentially a more restricted version of double object constructions in Levantine Arabic (Syrian below), which have a property that is critical for the analysis of Maltese that I describe first. In Syrian Arabic, some ditransitive verbs, such as *ʔaṭa* ‘give’, select two accusative complements, like English, shown in (37a), while other verbs, such as *baʔat* ‘send’, select a dative and an accusative object, like German, shown in (37b) (Cowell 1964, Hallman 2018). Dative Case is marked by the prefix *la‐* on the indirect object in (37b); accusative is unmarked.

**Table:** 

(37)	a.	ʔaṭeː‐t	Maːriya	l‐ktaːb.
		gave‐1sg	Maria	the‐book
		‘I gave Maria the book.’		
	b.	baʔat‐t	la‐Maːriya	l‐ktaːb.
		sent‐1sg	dat‐Maria	the‐book
		‘I sent Maria the book.’		

When pronominalized, a DP cliticizes to the local governor, here the verb. The clitic inflectional paradigm reflects the Case of the corresponding non‐pronominal argument, as illustrated in (38).

**Table:** 

(38)	a.	ʔaṭeː‐t‐a	l‐ktaːb.
		gave‐1sg‐her_ACC_	the‐book
		‘I gave her the book.’	
	b.	baʔat‐t‐illa	l‐ktaːb.
		sent‐1sg‐her_DAT_	the‐book
		‘I sent her the book.’	

Like English, both verb classes are compatible with a prepositional frame in which the recipient argument is expressed in an allative prepositional phrase and the theme functions as direct object, as shown in (39). In Arabic, the preposition is the same preposition *la‐* that marks dative Case, as a result of the cross‐linguistically common process of reanalysis of allative prepositions as dative Case markers (see Miyagawa and Tsujioka 2004 on a similar state of affairs in Japanese). But the clitic constructions in (38) must be derived from the double object frame in (37), not the prepositional frame in (39), since only in the former is the contrast between accusative and dative present that is reflected in the clitic inflections in (38).

**Table:** 

(39)	a.	ʔaṭeː‐t	l‐ktaːb	la‐Maːriya.
		gave‐1sg	the‐book	to‐Maria
		‘I gave the book to Maria.’		
	b.	baʔat‐t	l‐ktaːb	la‐Maːriya.
		sent‐1sg	the‐book	to‐Maria
		‘I sent the book to Maria.’		

Maltese shares the prepositional structure in (39) with Syrian Arabic, as shown in (40) (Sadler and Camilleri 2013, Camilleri et al. 2014, Maris Camilleri, p.c.).

**Table:** 

(40)	a.	Taj‐t	l‐ktieb	lil	Marija.
		gave‐1sg	the book	to	Maria
		‘I gave the book to Maria.’			
	b.	Bgħat‐t	l‐ktieb	lil	Marija.
		sent‐1sg	the‐book	to	Maria
		‘I sent the book to Maria.’			

But as Sadler and Camilleri (2013) point out, the double object construction corresponding to the Syrian examples in (37) is not grammatical in Maltese. Like the Romance languages that have influenced it considerably, Maltese does not seem to display a double object construction.

**Table:** 

(41)	a.	*Taj‐t	Marija	l‐ktieb.	
		gave‐1sg	Maria	the‐book	
		‘I gave Maria the book.’			
	b.	*Bgħat‐t	lil	Marija	l‐ktieb.
		sent‐1sg	dat	Maria	the‐book
		‘I sent Maria the book.’			

Yet, Maltese does display the constructional counterpart to the Syrian examples in (38), where the recipient argument is cliticized to the verb. As Sadler and Camilleri show, in this case the Case of the clitic varies with the choice of verb in the same way as in Syrian: accusative with *ta* ‘give’ and dative with *bagħat* ‘send’.

**Table:** 

(42)	a.	Taj‐t‐ha	l‐ktieb.
		gave‐1sg‐her_ACC_	the‐book
		‘I gave her the book.’	
	b.	Bgħat‐t‐ilha	l‐ktieb.
		sent‐1sg‐her_DAT_	the‐book
		‘I sent her the book.’	

In Syrian, the full DP recipient arguments in (37) show a Case distinction contingent on verb choice, a distinction that is preserved in the corresponding clitic constructions in (38). In Maltese, the clitic constructions corresponding to (38) are grammatical, and these, as in Syrian, cannot be derived from the prepositional frame shown in (40) (Syrian (39)) because the Case distinction is neutralized in the prepositional frame. Like the Syrian clitic construction, the Maltese clitic construction must be derived from a double object construction in which the recipient is differentially marked for Case depending on the choice of verb. But if the derivative clitic construction is grammatical in Maltese, why not the double object base for it? How does Maltese differ from Syrian such that the base for the clitic construction can surface as such in Syrian but not in Maltese?

What we are looking at in Maltese appears to be a very high definiteness requirement on what we might in the first instance call the ‘indirect object’, such that the indirect object must be pronominal (and pronouns cliticize to their governor in Maltese). This rules out (41) in Maltese because the indirect objects there are not pronominal. This restriction does not hold in Syrian. But ‘indirect objecthood’ cannot be associated with a particular Case in this language, since the high definiteness requirement holds of accusative and dative indirect objects alike. Further, the accusative that the indirect object of *ta* ‘give’ receives is the same Case as that borne by garden variety objects of transitive verbs. This is the Case that is withdrawn in passive, resulting in promotion to subject, illustrated in (43). Yet, accusative direct objects do not in general show the high definiteness requirement, as the examples above make clear.

**Table:** 

(43)	Marija	n‐għat‐at	il‐ktieb.
	Maria	pass‐gave‐3fs	the‐book
	‘Maria was given the book.’		

The high definiteness requirement also does not hold of a particular theta role: the recipient arguments in the prepositional construction in (40) need not be pronominal. Nor does the requirement hold simply of the first argument following the verb, which I referred to as the ‘primary object’ in section 2, because again, direct objects are not subject to the requirement, even when they are the initial object following the verb, as in the prepositional construction in (40). The requirement only holds of the first of two DP objects following the verb.

I claim that D‐licensing allows us to localize the restriction in Maltese configurationally. Suppose ΔP_2_—the higher of the two ΔPs that immediately dominate vP—imposes a very high definiteness requirement on the DP it licenses, such that that DP must be pronominal. In the double object construction, the three arguments (agent, recipient and theme) must be licensed by the three available ΔPs. Due to the Cyclic Linearization requirement, the second of the three arguments must be licensed by the second of the three ΔPs, meaning that this argument—the recipient—must be pronominal.

We predict that the recipient can escape this requirement when it has the option of being licensed by a ΔP other than ΔP_2_. In the prepositional construction in (40), for example, the recipient is Case‐ and D‐licensed within the PP, and so is not subject to the high definiteness requirement associated with ΔP_2_. The theme in this case may be licensed by either ΔP_2_ or ΔP_3_, but the option of being licensed by ΔP_3_, which does not carry the definiteness requirement, means it need not surface as a pronoun. A recipient argument may also escape the high definiteness requirement by being promoted to subject, as demonstrated in (43). When an accusative recipient is promoted to nominative in the passive, it is D‐licensed by the highest, sentence‐level ΔP_1_, which again does not impose a definiteness requirement.

I note here in passing that this analysis extends readily to Romance languages that appear to lack a double object construction. The fact that the underlying double object construction is the source of recipient clitics is particularly evident in Maltese because of the fortuitous coincidence that the Case of the indirect object in the double object construction varies according to choice of verb in that language, and this variation carries over to the clitic construction. While Romance languages do not offer this kind of evidence, Sportiche (2017) claims on the basis of evidence from binding patterns that indirect object clitics in ditransitive constructions in French are derived from a hidden double object frame. It is probably not a coincidence that Maltese has historically been in contact with Romance languages substantially more than Syrian Arabic.

The discussion above is intended to show two things. First, it presents another example where D‐licensing explains restrictions on the distribution of arguments that cannot be attributed to Case licensing. Just as in German, where Case requirements alone cannot explain the absence of secondary objects in adjectival passives, Case requirements alone cannot explain the requirement that the first of two objects be pronominal in Maltese. In both cases, D‐licensing offers a way of capturing the relevant restrictions. Second, the discussion of Maltese shows that, contra Chomsky's original notion of D‐licensing, there is arguably an at least tenuous connection between D‐licensing and definiteness after all. The connection is that on a language‐specific basis, a given Δ head might be specified to only license a strongly definite DP, where what counts as strongly definite is part of the specification of the individual Δ head.

## 9 Parameters of D‐Licensing

The observations about Maltese in the previous section complete a parameterization of the behavior of ΔPs in the three languages discussed here. I mentioned in section 6 that ΔP_1_ in English probes obligatorily and requires overt movement of the licensee to the specifier of ΔP_1_ (the EPP). Subjects in English may be indefinite, meaning ΔP_1_ does not place a definiteness requirement on its licensee. ΔP_2_ and ΔP_3_ also do not place a definiteness requirement on their licensee, but they probe only optionally and when they do probe, they do not require overt movement of the licensee. But in principle, a ΔP may trigger overt movement of its licensee optionally, deriving scrambling configurations in languages like German. Lastly, a ΔP may place a definiteness requirement on its licensee, like ΔP_2_ does in Maltese, though what counts as high definiteness might vary from language to language. Note too that there is apparently some degree of variation across languages in terms of whether Cyclic Linearization is an absolute requirement (English and Maltese) or a canonical preference (German). This range of variation is summarized below:

- ΔP probes obligatorily (ΔP_1_ in English) or optionally (ΔP_2_ and ΔP_3_ in English, all ΔPs in German).
- ΔP triggers overt movement of the goal obligatorily (ΔP_1_ in English) or optionally (all ΔPs in German) or not at all (ΔP_2_ and ΔP_3_ in English).
- ΔP licenses only DPs with a specified degree of definiteness (ΔP_2_ in Maltese, where the required degree of definiteness is ‘pronominal’).

It is typically the case, and on some accounts thought to be necessary, that syntactic features have some significance to the grammatical modules that interface with the syntax module. Case‐licensing, for example, has a clear functional utility: licensing connects the morphological form of the DP (its case inflection) to the syntactic relation that this inflection expresses (the DP's grammatical function, which is considered to have a configurational basis in generative linguistic theories). It is therefore natural to ask what the grammatical functional utility of D‐licensing is, and what role it plays in the modules that interface with syntax.

In the system postulated here, D‐licensing is obligatory for every DP, but the potential sensitivity to definiteness and the optionality of movement present the language with an opportunity to mark definiteness syntactically in the first case and to derive deviations from the neutral word order in the second, which in turn may be correlated with non‐neutral information packaging. Diesing (1992), for example, claims that the verb phrase (vP here) represents the semantic nuclear scope of the clause, whose content is asserted. Removal of DPs from the nuclear scope through scrambling in languages like German has the effect of placing those DPs in the portion of the clause whose content is presupposed, effectively shifting information from the base order into an order correlated with the difference between assertion and presupposition. Not all languages exploit this possibility. English, for example, lacks object shift. But D‐licensing as a mechanism can be put to use for these purposes.

It is arguably odd that D‐licensing is a requirement of DPs if its functional utility consists in deriving optional deviations from unmarked values of definiteness and word order. But in this respect, D‐licensing is similar to Case licensing, which is a requirement of DPs, yet is often unmarked, and when marked, often carries information structural implications. Enç (1991), for example, claims that overt object Case marking correlates with specificity in Turkish, and Browne (1970) and Karimi (1990) make similar claims for Persian. A body of literature on differential Case marking demonstrates the broad generalization that marked Case implies marked information structure (Bossong 1985, Comrie 1989, Croft 1990 and others). But not all languages avail themselves of the opportunity to differentially mark Case, and some languages do not mark Case overtly at all. Yet, as the conventional wisdom has it, all DPs must be Case licensed, even if the morphology is not overt. This state of affairs is very similar to what I described above for D‐licensing. I maintain, then, that all DPs must be Case‐ and D‐licensed. While some languages put the licensing mechanism to use in marking definiteness or information structure alternations, others, like English, make little use of this system, and unostentatiously license objects under Agree.

## 10 Verb Phrase Internal D‐Licensing

In the contemporary literature on verb phrase structure, only Bruening (2014) presents an analysis that seeks to exclude the derivation of adjectival participles with internal direct arguments in terms similar to those I have proposed. In this connection, Bruening treats a class of exceptions to the restriction on internal direct arguments in adjectival passives, and argues that the exceptional character of the verbs in question can be traced to their meaning. I claim that the D‐licensing analysis proposed here presents a better fit for the facts.

Bruening claims that examples like (44b), parallel to the examples in (19), are ruled out by a constellation of factors. First, in active double object constructions like (44a), the object DPs are attracted by little‐v and adjoin to vP for formal syntactic reasons (Bruening suggests Case‐licensing, though we would want to say something like D‐licensing to accommodate German). However, the feature of v that attracts these DPs must itself be licensed—‘activated’ in a sense—under c‐command by Infl. The adjectival participle forming operator interrupts this licensing relation. As a result, the DPs do not raise to vP in adjectival participles.

**Table:** 

(44)	a.	Jorge kicked Maria the ball.
	b.	*Maria is kicked the ball.

Crucial to Bruening's account is that ungrammaticality arises in (44b) as a result of a failure of semantic composition. The idea is that as in other accounts, the Appl‐VP complex denotes the *have* relation between recipient and theme, while little‐v denotes the *kick* relation between the agent and theme ((44a) means something like *Jorge's kicking the ball caused Maria to have the ball*). The theme must raise to vP in order to be mapped into the *kick* relation with the agent. The structure cannot be semantically composed if the theme fails to be attracted to v when the attracting feature of v is not activated because the adjective‐forming morpheme interrupts the licensing relation between Infl and v.

Bruening then points out that verbs like *deny*,* spare* and others are exceptions to the restriction evidenced in (44b), and relates their exceptionality to their semantic composition. The attested examples in (45) are representative (from Bruening's example (101), p. 401). Bruening also mentions *afford* in this connection; example (45c) is his example (103c), p. 401. A reviewer of the present work observes that *grant* also displays this behavior and provides the example in (45d).

**Table:** 

(45)	a.	Victim remains denied her American nationality…
	b.	He has a mass of gray‐black hair that appears spared the thinning that age usually inflicts on men.
	c.	…largely isolated from society, unafforded the opportunity to receive extensive formal education…
	d.	…all Mexican nationals who have entered the US legally and acquired a permanent residence permit will remain granted their permanent residence permit.

In these examples, the direct object occurs in its usual post‐verbal position within an adjectival participle, just what is typically excluded for ditransitive verbs. Bruening claims that the reason for this is that these verbs have a different internal semantic composition, one that does not require attraction of the object DPs to little‐v. He attributes the meaning in (46) to the verb *spare*. The agent is not represented in (46); it is added at the level of vP, which merges above the verb.

**Table:** 

(46)	[[spare]] = λxλf_<e,est>_λyλe . do(e) & ¬∃e′ [f(x)(y)(e′) & cause(e′)(e)]

This verb combines with an individual *x*, a relation *f*, and an individual *y* to derive a description of an event *e*. The relation *f* is the relation denoted by ApplP in the relevant example, i.e. *have*. The meaning of a sentence like (47a), then, comes out as in (47b).

**Table:** 

(47)	a.	The judge spared John the ordeal.
	b.	do(**the judge**, e) & ¬∃e′ [have(**John**,** the ordeal**, e′) & cause(e′)(e)]

In this case, the internal arguments are semantic arguments of the underlying verb, and none enters into a semantic relation with the agent in little‐v. In light of this, while formation of an adjectival participle prevents raising of the internal arguments to little‐v as before, this does not impact grammaticality because the derivation semantically composes without movement.

Bruening claims, then, that what makes examples like (45) grammatical is that they may semantically compose without raising of the theme to little‐v, just what the adjective forming operator makes impossible. But examples like *afford* and *grant* do not obviously differ from more pedestrian double object verbs like *give* in terms of their internal semantic composition. In fact, the composition attributed to *spare* in (46) does not yield quite the right truth conditions. There, causation is within the scope of negation so that the denotation in (47b) attributed to the sentence in (47a) reads ‘The judge did something that did not cause John to have the ordeal’. This is compatible with a situation in which John *did* have the ordeal, just not by virtue of anything the judge did. What we want is a semantic composition along the lines of what Beck and Johnson (2004) propose (p. 103, fn 4; they do not spell out the compositional details), where (47a) means that the judge caused John to not have the ordeal, schematized in (48), where John's not having the ordeal is the causee argument. This observation supports the garden variety cause‐to‐have structure attributed to transfer (or in this case withholding) of possession verbs by Harley (1997, 2002), Harley and Jung (2015), and others, rather than the structure in (47b).

**Table:** 

(48)	cause(**the judge**, [¬have(**John**,** the ordeal**)])

Further, Bruening's analysis hinges on the necessity of raising of both objects to v in the context of verbs whose adjectival participles do not allow internal direct arguments, and the impossibility of raising in the participle. As such, it requires raising of the object DPs to little‐v in cases where compositionality does not seem to demand it. The verb *give* often forms idioms with a direct object, as the examples in (49) show, modified from Larson (1988, p. 341).

**Table:** 

(49)	a.	The count gave Mary the creeps. (=made Mary feel uncomfortable)
	b.	Alice gave John hell. (=showed anger toward him)
	c.	Oscar gave Sue the boot. (=fired her)

Such examples do not seem to demand placing the direct object in a compositional semantic relationship with the external argument along the lines of what Bruening proposes for *kick*. While *John kicked Maria the ball* arguably involves John doing something to the ball, *The count gave Mary the creeps* does not involve the count doing anything to the creeps. Consequently, we would not want to place *the creeps* in a compositional semantic relation with little‐v, particularly since the agent, which is base generated in vP, is not part of the idiom. There should be no harm, then, in failing to raise the objects to v in idioms, yet *give* does not allow the theme to occur in its adjectival passive derivative, even in cases derived from (49). This observation suggests that the restriction on object licensing in adjectival participles is not due to failure of semantic composition.

**Table:** 

(50)	a.	*Mary is given the creeps.
	b.	*John looks given hell.
	c.	*Sue remains given the boot.

I propose instead that the verbs that allow a bare object in adjectival passives do so because they admit a ΔP projection within vP. Since it is unclear what semantic property characterizes the class of verbs in question, I assume pending further investigation that this availability is a lexical selectional specification of certain verbal roots. The internal structure for the adjectival participle *spared the ordeal* as in (51a), on this view, looks like (51b), where vP contains both a syntactic locus for negation (NegP) and for D‐licensing (ΔP).

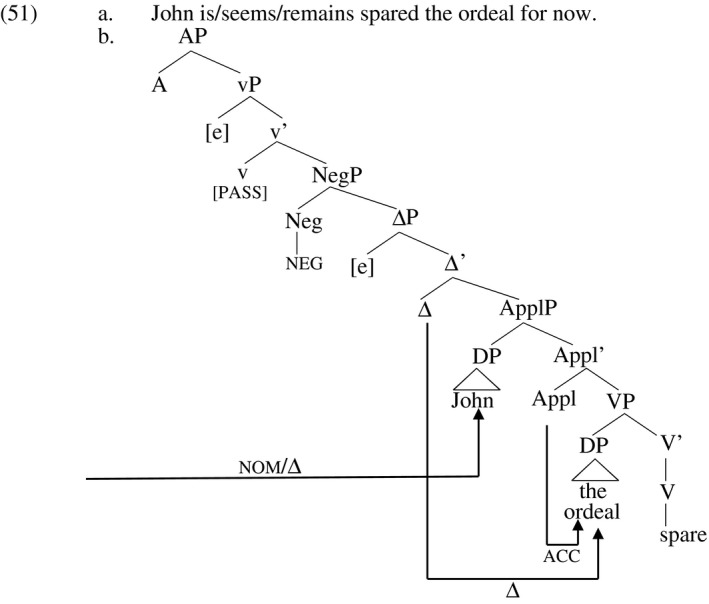

In this tree, the indirect object *John* receives nominative Case and D‐licensing from T and the adjacent Δ respectively (not shown), and the direct object *the ordeal* receives accusative Case from Appl and is D‐licensed by the local Δ, adjacent to Neg, internal to the participle. This analysis admits the existence of the exceptions that Bruening discusses as structures that admit vP‐internal ΔP. Whether this trait is connected to some other grammatical property or some semantic feature of the contexts involved remains to be investigated in greater detail.

## 11 Conclusion

In this article, I have endeavored to justify the hypothesis that DPs are subject not only to a Case licensing criterion but also an additional criterion, which I refer to as D‐licensing. It is this requirement that is unmet in adjectival passives of double object constructions. A purely Case‐theoretic analysis is not a feasible approach to this phenomenon for at least two reasons. First, many considerations point to the presence of vP in adjectival passives, leading to the expectation that secondary object Case licensing ApplP should be available too, unaffected by adjectival participle formation. Second, German adjectival participles are subject to the same restrictions as English even though the double object Case frame is different. German is particularly revealing, since the theme in verbal passive constructions receives Case not from ApplP but from outside the verb phrase (namely from T), meaning anything affecting vP or ApplP is not expected to affect assignment of nominative to the theme in adjectival passives. Here, something else must rule out the relevant cases. I have proposed that D‐licensing is the crucial factor. Further, D‐licensing provides a mechanism for capturing the otherwise puzzling requirement that the first object following the verb in Maltese (and possibly all languages that appear to lack a double object construction) must be pronominal when a secondary object follows. This requirement cannot be characterized as a requirement on a certain Case or a certain theta role, but its characterization as a requirement of ∆P_2_ captures the facts.

In this analysis, every DP must be licensed in an Agree relation to a ∆P. In some languages, this relation has a syntactic or semantic exponent: DP may raise overtly to the specifier of ∆P, and licensing may be contingent on a certain degree of definiteness in the DP. These conditions resemble those governing Case licensing. Overt Case marking may be optional, and its overtnes may be contingent on a certain degree of definiteness in the licensed DP. D‐licensing therefore is a process broadly similar to Case licensing. But the evidence reviewed in this work indicates that these are distinct processes. It is D‐licensing, not Case licensing, that fails in adjectival participles in English and German, and it is D‐licensing, not Case licensing, that is subject to a high definiteness requirement in Maltese. This work therefore teases apart two requirements on DPs that have previously been conflated.

## Notes

1 This example is grammatical on a habitual reading, which, however, strongly demands a licensing temporal quantifier, as in *Mary paints the door every spring*. A reviewer points out that eventive verbs may also have a property reading in the simple present, as in *Mary eats sushi*, meaning she is not predisposed to not eat sushi. I assume both cases are morphologically unmarked stative derivatives of the underlying eventive verb, and are therefore compatible with the simple present like other stative predicates.

2 Adjectival *un‐* differs from the ‘reversative’ *un‐* found in verbs like *uncover*. While the adjective *unpainted* means ‘not painted’, the verb *uncover* does not mean ‘to not cover’, but rather ‘to remove the cover’. Reversative *un‐* occurs in adjectival participles only if it is part of a verb that is adjectivized. There, it is compatible with adjectival *un*‐, as in *ununcovered*, meaning not having had the cover removed.

3 The recipient argument receives Case in its base position in German, before D‐licensing structure is merged, which is higher in the tree. At the point the recipient is merged, then, it becomes inactive for Case but remains active for D‐licensing. This disparity might play a role in ‘defective intervention’ configurations in languages such as Icelandic, in which a dative DP in certain configurations blocks agreement (but not Case) between a higher verb and a lower DP with structural nominative, yet does not itself control agreement (Chomsky 2000, Holmberg and Hróasdóttir 2004 and others). Such configurations do not arise in German (Broekhuis 2007), so I do not expand on the details here, except to intimate that in the relevant contexts in the relevant languages, the mechanism for agreement may be more closely syntactically connected to D‐licensing that to Case licensing.
